# How Do Safety Climate Ratings Relate to Attitudes Towards and Knowledge About Surgical Site Infection Prevention Measures?

**DOI:** 10.1017/ash.2021.145

**Published:** 2021-07-29

**Authors:** Yvonne Pfeiffer, Andrew Atkinson, Judith Maag, Michael Lane, David Schwappach, Jonas Marschall

## Abstract

**Group Name:** Watussi Study Group

**Background:** A positive safety climate is an important precursor of safe care outcomes. However, only limited evidence supports the association of low surgical-site infection (SSI) rates and positive safety climate. We investigated the role that perceptions of SSI prevention measures play for both safety climate level and strength as a subjective norm, that is, the social pressure perceived to perform the prevention measures, commitment to observe SSI prevention measures despite other situational pressures, and the level of knowledge about the prevention measures. **Methods:** The safety climate scale of the Safety Attitudes Questionnaire and 3 scales assessing subjective norm, commitment, and knowledge were used. All items were translated and retranslated from German to French and to Italian. All translated scales were pretested for understandability. Operating room (OR) personnel in 54 Swiss acute-care hospitals were surveyed, resulting in 2,769 analyzed responses with data aggregated on the hospital level. Two regression analyses were conducted: one using the percentage of positive responses per hospital as a safety climate level indicator, and another using the standard deviation of the safety climate ratings per hospital as a safety climate strength indicator. As independent variables, the hospital means of subjective norm, commitment, and knowledge were investigated and appropriately adjusted for number of respondents and sample composition. **Results:** The sample consisted of 1,495 nurses (54%) and 1,101 physicians (40%). Commitment and subjective norm were significant predictors (p < 0.001 and p < .05, respectively) of safety climate level, in the expected positive direction, but KNOW was not (R^2^, adjusted: 0.48); for safety climate strength, only COM was significant p < 0.001 (R^2^, adjusted: 0.27). **Conclusions:** The extent to which OR personnel were committed to perform the measures, such as timely administration of antibiotics, was associated with their safety climate rating level and strength. Thus, the rather general safety climate assessments are related to more specific safety behaviors necessary to achieve good outcomes such as low infection rates. Subjective norm was related to safety climate level only, indicating that in work environments with a good safety climate, the perceived social pressure to adhere to infection prevention measures may be higher. Knowledge about SSI prevention had no significant impact on safety climate, pointing to future research regarding the role of education in implementing prevention measures. Investigating how attitudes and knowledge about measures to prevent specific patient safety outcomes furthers our understanding of the role of safety climate in patient safety improvement.

**Funding:** No

**Disclosures:** None

